# Relationship between coping/attachment styles and infertility-specific distress in Iranian infertile individuals: A cross-sectional study

**DOI:** 10.18502/ijrm.v19i4.9061

**Published:** 2021-04-22

**Authors:** Forouzan Elyasi, Parisa Islami Parkoohi, Mahshid Naseri, Keshvar Samadaee Gelekolaee, Masoume Hamedi, Sepideh Peyvandi, Marzieh Azizi

**Affiliations:** ^1^Department of Psychiatry, Psychiatry and Behavioral Sciences Research Center, Sexual and Reproductive Health Research Center, Addiction Institute, School of Medicine, Mazandaran University of Medical Sciences, Sari, Iran.; ^2^Psychiatry and Behavioral Sciences Research Center, Addiction Research Institute, Mazandaran University of Medical Sciences, Sari, Iran.; ^3^School of Medicine, Mazandaran University of Medical Sciences, Sari, Iran.; ^4^Department of Reproductive Health, School of Nursing and Midwifery, Tehran University of Medical Sciences, Tehran, Iran.; ^5^IVF Ward, Imam Khomeini Hospital, Mazandaran University of Medical Sciences, Sari, Iran.; ^6^Sexual and Reproductive Health Research Center IVF Ward, School of Medicine, Mazandaran University of Medical Sciences, Sari, Iran.; ^7^Department of Reproductive Health and Midwifery, Nursing and Midwifery Faculty, Tehran University of Medical Science, Tehran, Iran.

**Keywords:** Infertility, Attachment, Copying, Distress.

## Abstract

**Background:**

Infertility has been recognized as a stressful clinical condition, significantly affecting couples' emotional functioning.

**Objective:**

To investigate the relationship between coping/attachment styles and infertility-specific distress (ISD) in infertile participants.

**Materials and Methods:**

Atotal number of 240 infertile participants (120 women and 120 men) who attend the Outpatient Infertility Clinic in Sari, Iran between February and October 2017 were selected using the convenience sampling method. Data were collected using a questionnaire addressing sociodemographic variables. In addition, coping and attachment styles were evaluated via the Coping Strategies Questionnaire and the Revised Adult Attachment Scale by Collins and Read (1990); respectively. Ultimately, the Infertility Distress Scale was used to assess ISD.

**Results:**

The mean ISD score was 42.53 ± 9.63. Secure and insecure attachment styles were observed in 37.9% and 62.1% of the cases, respectively. There was a significant difference among ISD and different groups of attachment styles (p = 0.001) and emotion-focused coping style (p = 0.021). However, no significant relationship was found between problem-focused coping style and ISD (p = 0.985).

**Conclusion:**

Considering the relationship between coping/attachment styles and ISD, it was recommended to implement stress prevention and coping education within the framework of coping/attachment theories for infertile individuals.

## 1. Introduction

Infertility, defined as a failure to conceive within 1 yr of unprotected sex (1). According to the World Health Organization, the worldwide prevalence rate of infertility is about 15% (2). Overall, 8% of Iranian couples are infertile (3). Infertility can negatively influence individuals' psychological functions. (4). In view of that, negative social attitudes may be directed toward infertile individuals. (5), since no control over one's life can be among the most problematic consequences of infertility (6). A bidirectional relationship has been thus far documented between infertility and distress. On the other hand, distress has been established as a major factor inflicting serious harm to general health status (7), as well as fertility performance (8). It has been reported that coping with distress can improve fertility in both women and men (9). Anxiety is an important psychological factor influencing infertility outcomes (10). In this respect, attachment refers to a relatively stable emotional bond between children and their mothers or other human beings interacting with children. Bowlby and colleagues have accordingly suggested that individuals' attachment styles could shape their adjustment approaches towards stressful experiences (11).

The three major attachment styles include secure, insecure-avoidant, and insecure-anxious/ambivalent (11). Individuals with different attachment styles seek to regulate their emotions and confrontations in such ways expressing their beliefs about themselves and others (12, 13). Attachment, as an individual characteristic, also affects infertile couples' mental and psychological reconciliation (14). Besides, studies have reported a significant relationships between individuals' attachment styles and infertility-specific distress (ISD) (1). Infertility has been correspondingly associated with anxiety and distress-induced avoidance in infertile people and even in those expecting infertility (1, 15).

According to Folkman and Lazarus, coping with stress is a psychological and behavioral effort to either overcome or tolerate stress or to minimize its effects (16). Coping strategies are comprised of two main styles. First, emotion-focused coping style that involves attempts to relieve negative emotional responses associated with distress such as fear, excitement, and frustration. Second, problem-focused coping style, that denotes psychological-based processing of actions and information (17). It has been established that infertility provokes distress. Because of their inability to control life events, decision-making problems, low self-esteem, and excessive stress, the emotion-focused coping style is more frequently exploited by infertile women (18). However, reportshave indicated close similarities between fertility- and infertility-coping strategies (19).

Overall, coping and attachment styles are key elements to understanding infertile couples' quality of life. The effectiveness of assisted reproductive techniques (ART) strategies is controversial due to higher propensity of individuals to psychological problems undergoing ART (20). Although, so far, numerous multidimensional studies have been conducted on ISD (21), most of the literature on this subject matter has addressed the effects of ART interventions on this type of distress (22) and a few studies have investigated relationships between coping/attachment styles and ISD (17, 18).

Therefore, the present study aimed to reflect on the relationship between Coping/Attachment Styles and ISD in Iranian Infertile Individuals.

## 2. Materials and Methods

This cross-sectional study was performed between February and October 2017 on infertile participants attending the Outpatient Infertility Clinic based in the city of Sari, Iran. The participants were selected using the convenience sampling method. With reference to the statistics of a similar previous study on infertile women in Iran (2), the initial sample size was determined by 97 individuals. Considering the 20% withdrawal rate, the sample size was increased to 120 individuals. Additionally, the results between the two independent groups of females and males were compared and the final sample size was set as 240 (120/gender).

### Inclusion and exclusion criteria 

The inclusion criteria were individuals who spoke Persian, had Iranian nationality, were diagnosed with primary infertility, and were willingness to participate in the study. Based on the present or past medical history, individuals with systemic diseases such as diabetes, high blood pressure, acute thyroiditis, and acute mental illnesses were excluded. In addition, individuals undergoing immunosuppressive therapy and those with a history of substance and alcohol abuse were removed. Remarriage was also considered as an exclusion criterion in the present study.

### Research tools

A sociodemographic characteristics information form, the Coping Strategies Questionnaire (CSQ), the Revised Adult Attachment Scale (RAAS) by Collins and Read (1990), and the Infertility Distress Scale (IDS) were used to collect the required information in this study.

### Sociodemographic characteristics information form

The questionnaire addressing the sociodemographic variables included queries about age, gender, weight, height, number of children, occupation, history of domestic violence, sexual harassment, physical punishment in childhood, history of sexually transmitted infections, satisfaction with marital life, and parental death during childhood. The participants were also asked if they were the main decision-makers regarding important life events or not. Psychotherapy history, level of education and income, duration of marriage, and duration of infertility, as well as the cause of infertility were further questioned.

### CSQ

The CSQ, initially developed by Lazarus and Folkman, evaluates eight dimensions including direct response, distance, self-control, social support, acceptance of responsibility, escape-avoidance, planned problem-solving, and positive re-evaluation. The given questionnaire consists of 66 items scrutinizing two main domains: emotion- and problem-focused coping styles. The four dimensions of social support, acceptance of responsibility, problem-solving, and positive re-evaluation are thus related to problem-focused coping style while the other dimensions are connected to the emotion-focused coping style (16). The scoring system is based on a four-point Likert-type scale (0 for “I have not used it at all,” 1 for “I have sometimes used it,” 2 for “I usually use it,” and 3 for “I always use it”). The questionnaire has been already validated by Rosenstiel and Keefe (0.71-0.85) (23). Furthermore, Jensen and Linton have confirmed the consistency of this questionnaire in all eight dimensions (Cronbach's alpha coefficients ranging from 0.60 to 0.90) (24). In another study by Rostami and colleagues, the Cronbach's alpha coefficient for this questionnaire has been reported as 0.87 (25).

### RAAS

The RAAS was primarily developed by Collins and Read (26). Theoretically, the given scale is based on the adult attachment theory. It includes 18 phrases wherein the respondents express the extent of their agreement or disagreement with these phrases on a five-point Likert-type scale. The three dimensions assessed by this scale include dependence (degree of reliance on others), closeness (level of intimacy and emotional closeness to others), and anxiety (degree of anxiety for being rejected and judged). Each of these dimensions is assigned with six phrases. The closeness dimension is comprised of phrases no. 1, 8, 9, 10, 14, and 17; the attachment dimension is made up of phrases no. 3, 4, 7, 15, 16, and 18; and the anxiety dimension is evaluated based on phrases no. 2, 5, 6, 12, and 13. Phrases no. 2, 3, 4, 9, 10, 16, 17, and 18 are reversely scored. To obtain the final score for each dimension, the scores of the phrases in each dimension are accumulated. Individuals achieving higher-than-the-mean scores in the closeness and the dependence dimensions and lower-than-the -mean score in the anxiety dimension are considered to have a secure attachment style. Accordingly, individuals obtaining higher-than-the-mean score in the anxiety dimension have an anxiety attachment style. Finally, individuals gaining higher-than-the-mean scores in all three dimensions are deemed to have avoidance attachment styles. The test-retest reliability indices for each dimension (i.e., closeness, dependency, and anxiety) have been reported as 0.68, 0.71, and 0.52, respectively (7). So far, the highest (0.74) and the lowest (0.28) reliability scores have been obtained for the anxiety and attachment dimensions among Iranians, respectively. In addition, the reliability score of the closeness dimension has also been moderate (0.52), similar to the results of the tes- retest method (27).

### IDS

The IDS was developed by Akyüz and colleagues in their study on Turkish infertile women in 2008 (28). This scale consists of 21 items scored on a four-point Likert-type scale [i.e., never (1), sometimes (2), often (3), and always (4)]. The achievable score ranges from 21 to 84 with higher scores indicating higher levels of distress. Item no. 3, 10, 13, 14, and 21 are also reversely scored. Arab-Sheybani and colleagues validated the IDS for being administered in Iran (Cronbach's alpha coefficient of 0.91) in 2010 (29).

### Ethical considerations

This study was approved by the Ethics Committee of the Mazandaran University of Medical Sciences (Ethical code: IR.MAZUMS.REC.1396.2294). In addition, a written informed consent was signed by all participants.

### Statistical analysis

The statistical analysis was performed using the SPSS (Statistical Package for the Social Sciences SPSS, version 17.0, SPSS Inc., Chicago, Illinois, USA). The sociodemographic variables as well as the attachment and coping styles were presented using descriptive statistics. The normal distribution of the data was further evaluated by histograms, distribution charts, and the Kolmogorov-Smirnov test. The quantitative variables including distress score and coping styles followed a normal distribution. They were also presented by means and standard deviations (SDs). The one-way analysis of variance (ANOVA) was additionally utilized to compare levels of distress between different attachment styles. The levels of distress in men and women were then compared using the independent-samples Student's *t* test. The correlation between distress and the problem-solving styles was additionally assessed using the Pearson's correlation coefficient. As no significant linear relationship was detected between the independent (i.e., coping styles) and the dependent variables, the linear regression model was not applied. The significance level was considered at p < 0.05.

## 3. Results

### Participants' sociodemographic characteristics 

A total number of 240 infertile individuals were recruited in the present study, with a mean age of 33.23 ± 6.85 yr ranging from 18 to 61 yr; the mean age of female and male participants being 32.00 ± 6.51 and 34.47 ± 6.98 yr; respectively. While the mean infertility duration was 6.06 ± 5.02 yr (ranging from 1 to 28 yr), the mean number of children was 4.96 ± 2.06. Majority (49.2%) of the participants had already used ART. Overall, 213 (88.8%) individuals were participating in the decision-making process in their lifetime. In addition, approximately 10% of the participants had lost their mothers, fathers, or both in childhood (Table I).

### Attachment styles

In this study, 37.9% of the couples represented a secure attachment style. Likewise, 29.6%, 22.1%, and 10.4% of them demonstrated anxious, avoidant, and pre-occupied attachment styles, respectively. In general, the insecure attachment styles were predominant in both women (62.1%) and men (63.3%) in the present study (Table II and III). The Chi-square test also showed similar frequency distributions in the four sub-categories between men and women. In other words, the attachment styles were independent of gender (p = 0.613).

### Coping styles

Table III presents the distribution of coping styles in infertile participants.

### ISD

The mean ± SD score was 42.53 ± 9.63. Furthermore, the mean scores of infertility distress were similar in men (42.50 ± 9.5) and women (42.55 ± 9.7) (mean difference = 0.05, 95% confidence interval [CI]: -2.404 to -2.504, p = 0.968).

### Relationship between ISD and sociodemographic variables

The levels of ISD were significantly different among the different levels of income, age groups, and in women. Such differences were related to the comparisons between age groups < 29 and > 50 yr, as well as between those of 30 and 39, and 40 and 49 yr. On the other hand, no statistically significant difference was observed in the levels of ISD comparing the different age groups in infertile men. Also, there were no significant differences in the ART groups (Table IV).

### Relationship between ISD and coping styles 

No statistically significant relationships were seen between ISD and problem-focused coping style neither in general (p = 0.98) nor in women (p = 0.765) and men (p = 0.827). However, a statistically significant relationship was observed between ISD and emotion-focused coping style in total (p = 0.021) and in men (p = 0.259, Table V).

### Relationship between ISD and attachment styles

**Figure 1:**
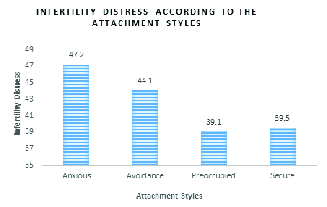
Infertility distress according to the attachment styles.

The levels of ISD were significantly different among individuals with various attachment styles. According to Scheffe's method as a post-hoc test, the differences were observed in individuals with secure versus avoidant, secure versus anxious, and preoccupied versus anxious attachment styles (Figure 1). No significant differences were observed between the levels of ISD in both men and women with different attachment styles. Among women, the differences were observed in individuals with secure versus anxious, busy versus avoidant, and busy versus anxious attachment styles. In men, a significant difference was found in the levels of ISD comparing those with secure versus anxious attachment styles (Table VI).

**Table 1:** Sociodemographic, psychological, and fertility-related medical history in infertile participants


	**Female (n = 120)**	**Male (n = 120)**
**Educational status**			
	**Illiterate**	7 (5.8)	9 (7.5)
	**Pre-academic education**	81 (67.6)	81 (67.5)
	**Academic education**	32 (26.6)	30 (25)
**Job**			
	**Housewife**	102 (85)	1 (0.8)
	**Worker**	4 (3.3)	49 (40.8)
	**Employee**	5 (4.2)	14 (11.7)
	**Free job**	7 (5.8)	50 (41.7)
	**Others**	2 (1.7)	6 (5)
**Family income (Rials)**			
	**5000000-10000000**	79 (65.8)	82 (68.3)
	**10000000-15000000**	32 (26.7)	27 (22.5)
	**15000000-20000000**	6 (5)	8 (6.7)
	**> 20000000**	3 (2.5)	3 (2.5)
**Infertility causes**			
	**Female factor**	22 (18.3)	22 (18.3)
	**Male factor**	35 (29.2)	34 (28.3)
	**Both**	23 (19.2)	26 (21.7)
	**Unexplained/unclear**	40 (33.3)	38 (31.7)
**Assisted reproductive technology**			
	**Fertility medication**	58 (48.3)	60 (50)
	**ICSI**	30 (25)	29 (24.1)
	**In vitro fertilization**	23 (19.2)	21 (17.5)
	**Egg donation**	4 (3.3)	5 (4.2)
	**Follow-up**	3 (2.5)	3 (2.5)
	**ZIFT**	2 (1.7)	2 (1.7)
**Sexually transmitted disease**			
	**Yes**	30 (25)	9 (7.5)
	**No**	90 (75)	111 (92.5)
**Marital satisfaction**			
	**Very low**	2 (1.7)	2 (1.7)
	**Low**	2 (1.7)	1 (0.8)
	**Moderate**	28 (23.3)	14 (11.7)
	**High**	33 (27.5)	44 (36.7)
	**Very high**	55 (45.8)	59 (49.1)
**Domestic violence**			
	**Yes**	0 (0)	24 (20)
	**No**	120 (100)	96 (80)
**Physical abuse**			
	**Yes**	9 (7.5)	23 (19.2)
	**No**	111 (92.5)	97 (80.8)
	**Person himself/herself**	5 (4.2)	11 (9.2)
	**Spouse**	8 (6.7)	2 (1.7)
**Parental death in childhood**			
	**Yes**	6 (5)	7 (5.8)
	**No**	114 (95)	113 (94.2)
**Psychological counseling history**			
	**Yes**	20 (16.7)	12 (10)
	**No**	100 (83.3)	108 (90)
Data presented as n (%). ICSI: Intra cytoplasmic sperm injection, ZIFT: Zygote intrafallopian transfer			

**Table 2:** Distribution of attachment styles in infertile participants


	**Total (n = 240)**	**Female (n = 120)**	**Male (n = 120)**
**Attachment style**				
	**Secure**	91 (37.9)	47 (39.1)	44 (36.7)
	**Insecure**	149 (62.1)	73 (60.9)	76 (63.3)
**Insecure attachment style**				
	**Preoccupied**	25 (10.4)	15 (12.5)	10 (8.3)
	**Dismissive-avoidant**	71 (29.6)	32 (26.7)	39 (32.5)
	**Fearful-avoidant**	53 (22.1)	26 (21.7)	27 (22.5)
Data presented as n (%)				

**Table 3:** Distribution of coping styles in infertile participants and differences between men and women regarding emotion- and problem-focused coping styles


**Variables**		**Total**	**Women**	**Men**	**P-value**
**Problem-solving focused coping styles**		37.37 ± 9.54	38.08 ± 8.51	36.66 ± 10.46	0.248*
	**Social support**	9.88 ± 3.54	10.49 ± 3.21	9.27 ± 3.76	0.007*
	**Acceptance of responsibility**	6.42 ± 2.41	6.53 ± 2.30	6.31 ± 2.52	0/868**
	**Planned problem-solving**	9.83 ± 3.25	9.81 ± 3.01	9.86 ± 3.48	0.906*
	**Positive re-evaluation**	11.24 ± 3.04	11.25 ± 2.79	11.23 ± 3.29	0.892**
**Emotion-focused coping styles**		38.21 ± 9.67	38.11 ± 9.47	38.32 ± 9.91	0.868*
	**Direct response**	8.17 ± 8.00	7.89 ± 2.87	8.45 ± 3.06	0.066**
	**Distance**	9.00 ± 3.27	9.35 ± 3.27	8.65 ± 3.24	0.098*
	**Escape-avoidance**	9.84 ± 3.88	9.44 ± 3.89	10.23 ± 3.84	0.114*
	**Self-control**	11.20 ± 3.32	11.43 ± 3.06	10.98 ± 3.57	0.305*
All data presented Mean ± SD, *Independent *t* test, **Mann–Whitney U-test					

**Table 4:** Description and comparison of ISD scores regarding different sociodemographic and ART groups in 240 infertile cases


		**95% confidence interval for mean**		
<brow>-2</erow> **Variable**	<brow>-2</erow> **Frequency (n = 240)**	<brow>-2</erow> **Mean ± SD**	**Lower bound**	**Upper bound**	<brow>-2</erow> **Value of F/t/U**	<brow>-2</erow> **P-value**
**Gender**						
**Male**	120	42.50 ± 9.59		
**Female**	120	42.55 ± 9.70	<brow>-2</erow> –2.404	<brow>-2</erow> 2.504	<brow>-2</erow> 237.96	<brow>-2</erow> 0.968${}^{**}$
**Age groups**						
**Lower 29**	76	42.80 ± 9.101	40.72	44.88	3.350	0.020${}^{*}$
**30–39**	119	43.80 ± 9.567	42.06	45.53	
**40–49**	38	38.26 ± 9.901	35.01	41.52	
**Upper 50**	7	41.00 ± 10.182	31.58	50.42	
**Income**						
**5000000–10000000**	161	44.13 ± 9.424	42.65	45.61	
**10000000–15000000**	59	40.00 ± 9.372	37.56	42.44	
**15000000–20000000**	14	38.50 ± 10.204	32.61	44.39	
**> 20000000**	6	35.83 ± 8.495	26.92	44.75	<brow>-4</erow> 3.674	<brow>-4</erow> 0.006${}^{{}^{''}}$
**ART**						
**Fertility medication**	118	9.817 ± 0.904	39.95	43.53	
**ICSI & microinjection**	63	9.727 ± 1.225	41.04	45.94	
**In vitro fertilization**	44	9.514 ± 1.434	40.49	46.28	
**Egg donation & embryo donation**	15	8.305 ± 2.144	37.53	46.73	<brow>-4</erow> 0.597	<brow>-4</erow> 0.617*
*One-way ANOVA, **t test, ISD: Infertility-specific distress, ART: Assisted reproductive techniques, ICSI: Intra cytoplasmic sperm Injection						

**Table 5:** Mean and standard deviation of coping styles and relationship between coping styles and ISD in infertile individuals


	**Coping styles**				**ISD**					
			**Total**		**Female**		**Male**	
	**Total**	**Female**	**Male**	**P-value**	**CC**	**P-value**	**CC**	**P-value**	**CC**	**P-value**
**Problem- focused coping strategy**	37.37 ± 9.54	38.08 ± 8.51	36.66 ± 10.46	0.248	–0.001	0.985	–0.028	0.765	0.020	0.827
**Emotion-focused coping strategy**	38.21 ± 9.67	38.11 ± 9.47	38.32 ± 9.91	0.868	0.149	0.021	0.104	0.259	0.193	0.035
Data presented as Mean ± SD, Pearson's correlation coefficient, CC: Correlation coefficient, ISD: Infertility-specific distress, SD: Standard deviation										

**Table 6:** Description and comparison of ISD scores regarding different attachment styles in 240 infertile cases


	**95% Confidence interval for mean**		
<brow>-2</erow> **Attachment styles**	<brow>-2</erow> **Mean ± SD**	**Lower bound**	**Upper bound**	<brow>-2</erow> **Value of F**	<brow>-2</erow> **P-value**
**Secure (91)**	39.55 ± 9.01	37.67	41.43	
**Fearful (n = 53)**	47.30 ± 9.07	44.80	49.80	
**Dismissive (n = 71)**	43.89 ± 9.25	41.70	46.08	
**Preoccupied (n = 25)**	39.36 ± 9.53	35.42	43.30	<brow>-4</erow> 9.540	<brow>-4</erow> 0.001*
ISD: Infertility-specific distress,* One-way ANOVA					

## 4. Discussion

### Relationship between ISD and sociodemographic variables

In this study, conducted on 240 infertile participants, the mean score of ISD was obtained as 42.53 ± 9.63 (range 21-48) with higher infertility scores representing higher levels of distress. Of note, loss of self-esteem, sexual distress, depression, sense of guilt, anxiety, frustration, emotional distress, and communication problems are commonplace among infertile couples (30, 31). In a study by Peyvandi and colleagues, the Cattle Anxiety scale had been further used to examine anxiety and distress in infertile individuals (32). While they had reported no anxiety in 50.5% of their participants, mild, moderate, severe, and very severe anxieties had been described in 19%, 17.5%, 11%, and 2% of the participants, respectively (32). In a cross-sectional study in Turkey on 100 infertile women and 100 healthy controls, the mean anxiety and depression scores had been slightly but not significantly higher in infertile women than fertile partners (33). Furthermore, infertile women had represented greater physical and psychological disabilities and poorer quality of life (33).

In the present study, the highest and the lowest levels of ISD were observed in the age groups of 30-39 and 40-49 yr. Likewise, there were statistically significant differences in the levels of ISD between different age groups in infertile women but not in men, indicating higher ISD in younger women. This might be due to lower life experience, lower ability to adapt to infertility, as well as higher distress toward early pregnancies in younger women. But in another study about factors associated with infertility distress of infertile women, no statistically significant linear relationship was found between the woman's age and the IDS score. (34). According to another survey in Turkey, self-reported disability had been higher among young infertile women (33). Nevertheless, no significant differences had been reported comparing ISD between different age groups in the study by Ramos and colleagues (35).

In the present study, no statistically significant difference was detected between men and women in terms of ISD. These findings were in line with the investigation by Donarelli and colleagues (22). In addition, Alosaimi and colleagues reported ISD in 39.7% of infertile males and 47.3% of infertile females (36). These findings suggested that both women and men could be equally susceptible to ISD and its related problems. Although the differences in ISD were not statistically significant between men and women, this did not necessarily mean similar structures of infertility in males and females. In fact, infertility can be considered as an indicator of poor masculinity performance as well as a social stigma in men (37). Another study noted that men could more commonly suffer from disturbing affairs such as being encouraged to remarry or get divorced, while women were more likely to be burdened with mental and emotional exhaustion, marital conflicts, unfavorable attitudes of their spouses' relatives or society, and constant desire of their spouse for a child (36).

Besides, there was a significant relationship between family income and ISD. In this regard, families with lower income represented higher ISD. These findings contrasted with the results reported in the study by Orlitzky and colleagues who described no significant relationship between levels of income and ISD (38). This disagreement might be in part explainable by the fact that most participants in the recent study had moderate income. Given the fact that infertility therapeutic modalities are costly, poor financial status may exaggerate ISD.

### Relationship between coping styles and ISD

Overall, a significant relationship was identified between ISD and emotion-focused coping style. The higher levels of ISD in individuals with emotion-focused coping styles might be due to failure to control one's life events as well as high psychological pressures in those adopting this style. Furthermore, ART interventions may exaggerate ISD in couples exploiting emotion-focused coping style because of its aggressive and stressful nature and high failure rate. However, there was no significant relationship between emotion-focused coping style and ISD in women. This was in conflict with the report by Tamannaifar and colleagues who described significant differences between mental health status, marital adjustment, and coping responses in infertile women and their fertile partners. The infertile women also demonstrated poorer mental health and marital adjustments in comparison with fertile women in a recent study (39).

In the present study, no statistically significant relationship was found between problem-focused coping style and ISD in both genders. Regarding different dimensions of problem-focused coping styles, women acquired a higher mean score than men in seeking social support. However, there were no significant differences in the mean scores of planned problem-solving, accountability, positive re-evaluation, direct response, avoidance, escape-avoidance, and self-esteem domains between women and men. In comparison, women with strong avoidance coping styles had experienced lower levels of ISD in the study by Aflak-Sair and colleagues (40). In a recent report, regression analysis had further shown that avoidance-active (B = 0.35, p < 0.001) and meaning-based (B = 0.50, p < 0.001) coping styles had significantly predicted ISD (40). In another study by Cassidy and colleagues, women using problem-solving and affective-based coping styles had felt lower sense of guilt and higher security in their relationships (41).

### Relationship between attachment styles and ISD

In this study, 37.9% of the participants demonstrated a secure attachment style. In addition, 62.1%, 29.6%, 22.1%, and 10.4% of the participants represented insecure, anxious, avoidant, and busy attachment styles, respectively. In addition, 39.2% and 60.8% of infertile women used secure and insecure attachment styles, respectively, while 36.7% and 63.3% of men, respectively, showed secure and insecure attachment styles. In another study on infertile couples, 58.7% and 63% of women and men had, respectively, employed secure attachment style (14). According to the observations in the present study, different attachment styles were significantly associated with ISD in total and in women. According to the Scheffe's method as a post-hoc test, ISD was significantly different, comparing individuals with secure and anxious, busy and avoidant, as well as busy and anxious attachment styles. Accordingly, the highest level of ISD was related to the participants with anxious attachment style, while the lowest level was observed in individuals with a secure attachment style. Of note, secure attachment styles can effectively boost the ability to accommodate with stressful conditions such as infertility. This can explain the lower level of distress in infertile couples with a secure attachment style. In the study by Donarelli and colleagues, the dimensions of attachment style (i.e., anxiety and avoidance) significantly correlated with ISD (22). There had been significant relationships between anxiety and ISD and avoidance dimensions of attachment style. Accordingly, women exploiting avoidance attachment style had higher levels of infertility distress, as well as higher sexual and communication concerns. Likewise, men utilizing avoidance attachment represented higher levels of anxiety and ISD, as well as higher social, sexual, and communication concerns (35). In the study by Talebi and colleagues, significant relationships had been described between marital conflicts and attachment styles as those applying doubtful coping style had suffered from more marital conflicts than those with either secure or insecure attachment styles. However, no significant differences had been reported in the marital conflicts regarding secure and avoidance attachment styles (42). In their study on 275 women with primary infertility, Besharat and colleagues had further shown that secure attachment style was related to higher coping ability in infertile women (43). This is while avoidant and ambivalent attachment styles had predicted weaker coping abilities in a recent report (43). This was similar to the findings of the present study regarding a significant relationship between anxiety and avoidance dimensions of attachment style and ISD.

### Limitations

As one of the major limitations of this study, no control group (i.e., fertile couples with children) was incorporated. Other limitations included small sample size, unavailability of participants' medical history, administration of questionnaires instead of interviews for data collection, and use of a single-center study.

### Implications for practice

The findings of this study can be used by healthcare staffs working in infertility clinics. Physicians and mental healthcare professionals can also exploit the findings of this study to evaluate attachment styles. While short-term IVF treatments may not provide individuals with the opportunity to change their basic attachment patterns, they can help couples understand how their attachment behaviors affect ISD. Psychological interventions such as spousal counseling sessions can be further recruited to manage ISD in couples undergoing IVF. Ultimately, educating infertile couples on how to accommodate the conditions and to seek support may help alleviate the negative consequences of infertility.

### Implications for research

Infertility can significantly affect sexuality and marital relationships and increase individuals' distress. Further studies are accordingly recommended to scrutinize the impacts of sexual functionality on ISD. In addition, longitudinal research is recommended to investigate the effects of counseling and attachment sessions on ISD. It is also applicable to assess the influence of different infertility etiologies on ISD in further studies.

## 5. Conclusion

Considering the relationship between coping/attachment styles and ISD, it is recommended to implement stress prevention and coping education within the framework of coping/attachment theories for infertile individuals.

##  Conflict of Interest

None declared.
